# Swimming kinematics of rainbow trout behind a 3×5 cylinder array: a computationally driven experimental approach to understanding fish locomotion

**DOI:** 10.1242/jeb.247873

**Published:** 2024-12-05

**Authors:** David Sparks, Edwin Rajeev, Subhra Shankha Koley, Alberto Canestrelli, James C. Liao

**Affiliations:** ^1^ Department of Biology, Whitney Laboratory for Marine Bioscience, University of Florida, St Augustine, FL 32080, USA; ^2^ Department of Neuroscience, University of Florida, Gainesville, FL 32610, USA; ^3^ Department of Civil and Oceanic Engineering, University of Florida, Gainesville, FL 32611, USA; ^4^ Cummins Cederberg - Coastal & Marine Engineering, Miami, FL 33134, USA; ^5^ Southwest Research Institute (SwRI), San Antonio, TX 78238-5166, USA

**Keywords:** Computational Fluid Dynamics, Fish, Particle Image Velocimetry, Hydrodynamics, Locomotion

## Abstract

Fish in the wild often contend with complex flows that are produced by natural and artificial structures. Research into fish interactions with turbulence often investigates metrics such as turbulent kinetic energy (TKE) or fish positional location, with less focus on the specific interactions between vortex organization and body swimming kinematics. Here, we compared the swimming kinematics of rainbow trout (*Oncorhynchus mykiss*) holding station in flows produced by two different 3×5 cylinder arrays. We systematically utilized computational fluid dynamics to identify one array that produced a Kármán vortex street with high vortex periodicity (KVS array) and another that produced low periodicity, similar to a parallel vortex street (PVS array), both validated with particle image velocimetry. The only difference in swimming kinematics between cylinder arrays was an increased tail beat amplitude in the KVS array. In both cylinder arrays, the tail beat frequency decreased and snout amplitude increased compared with the freestream. The center of mass amplitude was greater in the PVS array than in only the freestream, however, suggesting some buffeting of the body by the fluid. Notably, we did not observe Kármán gaiting in the KVS array as in previous studies. We hypothesize that this is because (1) vorticity was dissipated in the region where fish held station or (2) vortices were in-line rather than staggered. These results are the first to quantify the kinematics and behavior of fishes swimming in the wake of multiple cylinder arrays, which has important implications for biomechanics, fluid dynamics and fisheries management.

## Teaser

**Summary:** The swimming kinematics of rainbow trout are largely preserved across two, 3×5 cylinder array treatments that differ in vortex periodicity and turbulent kinetic energy.

## INTRODUCTION

Fishes living in currents commonly hold station in the complex flows found behind both natural objects such as rocks, corals and submerged vegetation, and man-made structures such as bridge pilings and dams. The cost of swimming in these flows varies: some studies show that fish exhibit higher oxygen consumption and decreased stability in turbulence (Enders et al., 2003; Tritico and Cotel, 2010; Webb, 1998). Other studies show that fish that hold station around a bluff body consume less oxygen compared with swimming in uniform flow (Liao et al., 2003a,b; Przybilla et al., 2010; Taguchi and Liao, 2011). Kármán gaiting fish, for example, exploit the staggered, alternating vortices of a vortex street by slaloming between them (Liao, 2004; Liao et al., 2003b). Entraining and bow waking fish exploit local high-pressure regions around a cylinder to perform minimal swimming movements (Liao et al., 2003a; Przybilla et al., 2010).

Despite the prevalence of assemblages of bluff bodies in the current-swept environments inhabited by fishes, very little is known about the effect of their downstream wakes on the mechanics of fish swimming. Yet, extending our understanding of environmental energy recapture into more physically complex habitats promises to provide new insights and strategies for efficient fish locomotion (Liao, 2022) and inform the design of fishway passages to minimize the impact on native ecosystems (Castro-Santos et al., 2009; Puzdrowska and Heese, 2019; Lacey et al., 2012; Wilkes et al., 2017). A fuller understanding of how fishes can benefit energetically from swimming in these complex habitats will require a discrete examination of the details of the flow structure that fish depend on, such as vortex size or periodicity (Liao et al., 2003a,b; Enders et al., 2003; Stewart et al., 2016).

A great diversity of wakes can be produced behind multiple cylinders based on the ratio of streamwise to cross-stream cylinder gap space (Gao et al., 2020). We therefore ran computational fluid dynamics (CFD) simulations on 60 cylinder arrays of differing spacing ratios to search the parameter space of wakes based on vortex shedding periodicity (Stewart et al., 2016). From this search, we selected wakes from two different cylinder arrays. The first array exhibited a discrete Kármán vortex street, characterized by high vortex periodicity and is herein called the KVS array. This vortex street is similar to the alternating vortex street seen in single-cylinder vortex shedding (Blevins, 1990; Liao et al., 2003a,b). The second array exhibited low vortex shedding periodicity, resembling a parallel vortex street (Karasudani and Funakoshi, 1994), and is herein called the PVS array. This vortex street is similar to the symmetric vortex streets observed in cylinders in confined flows (Ngyuen et al., 2018).

This study employed a combination of systematically selected CFD simulations with live experiments on rainbow trout to directly test the effects of specific wake parameters on station-holding swimming kinematics.

## MATERIALS AND METHODS

### Overview

**Fig. 1.**
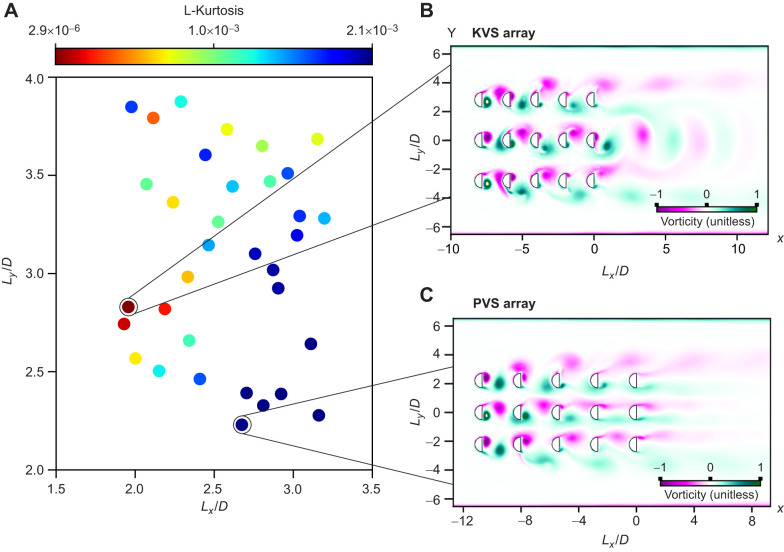
**Periodicity optimization study results of computational fluid dynamics (CFD) simulations that lie within the co-shedding regime.** (A) The values of the objective function [L-kurtosis of the fast Fourier transform (FFT) spectrum] plotted against the spacing ratio (*L_x_*/*D* and *L_y_*/*D*, where *L_x_* and *L_y_* are the ratio of cylinder gap space in the streamwise and in the cross-stream directions, respectively, to cylinder diameter *D*) identified from an initial sample size of 60 simulations. (B) Optimal [Kármán vortex street (KVS) array] and (C) sub-optimal [parallel vortex street (PVS) array] vorticity contours selected for the experiment. Color gradient scale represents vorticity (s^−1^). The experimental cylinder array was constructed based on these two arrangements. (*x* and *y* axes in *L_x_*/*D* and *L_y_*/*D*, respectively.)

Rainbow trout, *Oncorhynchus mykiss* (Walbaum 1792), were exposed to two cylinder arrays, as well as a control treatment with no cylinders (freestream flow). Each of the three treatments was run at three flow speeds (22, 48 and 74 cm s^−1^) in a 175 l recirculating flow tank (Loligo Systems, Tjele, Denmark). CFD simulations for one cylinder array predicted a highly periodic flow with a discreet Kármán vortex street, called the KVS array (Fig. 1B). Simulations for the other cylinder array predicted a low periodic flow, which included similar features to a parallel vortex street, called the PVS array (Fig. 1C). Simulation parameters were validated using particle image velocimetry (PIV). A high-speed camera captured images of the ventral silhouette of fish at 100 frames s^−1^ (Phantom Miro 340, Vision Research, Wayne, NJ, USA). A machine learning program (DeepLabCut) was then trained on 260 annotated frames at 600,000 iterations to recreate the outline of the fish in each frame. Using customized MatLab scripts, fish midlines were then reconstructed from these outlines and used to calculate body kinematics such as tail beat frequency, body wavelength, snout amplitude, center of mass (COM) amplitude and tail tip amplitude (Liao et al., 2003a).

### Hydrodynamic treatment selection

**Fig. 2.**
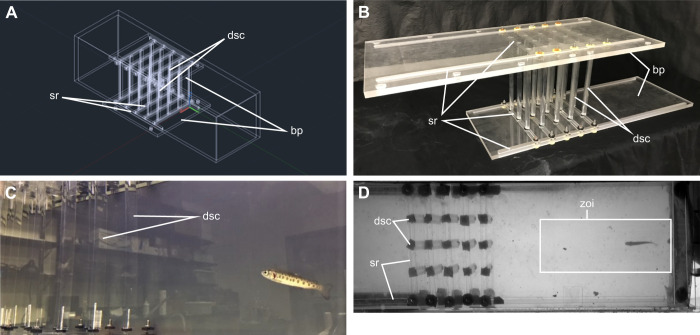
**Experimental set up.** (A) Computer aided design (CAD) drawings of parts for an adjustable array of multiple D-shaped cylinders were designed using SolidWorks. (B) Parts were laser cut from clear acrylic and cylinders were purchased separately. Arrays of D-shaped cylinders were manually constructed to replicate both CFD simulations of cylinder arrays within ±1 mm (appears circular because of the use of washers). (C) Five rainbow trout (*Oncorhynchus mykiss*, length 7.4±0.1 cm) each individually swam downstream from each combination of hydrodynamic treatments (freestream, Kármán vortex street, parallel vortex street) and flow speeds (22, 48 and 74 cm s^−1^). (D) High-speed video recordings of 16 tail beats of the ventral silhouette were taken for each individual×hydrodynamic treatment×flow speed combination within a pre-determined zone of interest. dsc, D-shaped cylinder; sr, sliding rail; bp, baseplate; zoi, zone of interest.

Two cylinder arrays were selected based on the wakes predicted across 60 simulations (Fig. 1A): (1) a highly periodic flow with a discreet Kármán vortex street (KVS array; *L_x_*/*D*=1.9, *L_y_*/*D*=3.0, where *L_x_* and *L_y_* are the ratio of cylinder gap space in the streamwise and in the cross-stream directions, respectively, to cylinder diameter *D*; Fig. 1B) and (2) a low periodic flow with similar features to a parallel vortex street (PVS array; *L_x_*/*D*=2.6, *L_y_*/*D*=2.2; Fig. 1C).

#### Computational fluid dynamics

2D flow fields were modeled around D-shaped cylinder arrays using CFD in OpenFOAM (v2012). The CFD model numerically solved the Navier–Stokes equations that govern incompressible fluid motion and a 2D shear stress transport (SST) *k*-ω unsteady Reynolds averaged Navier–Stokes (URANS) turbulence model was adopted.

A finite volume method with an overset grid solved the incompressible Navier–Stokes equations, which accounts for maintaining constant momentum. The walls of the half-cylinders were discretized using a structured grid and divided into 130 grid points. Because of the complex nature of vortex interactions between cylinders inside an array, turbulence was fully resolved at the cylinder wall. The effects of the tank side walls were also taken into account for the cylinder–vortex interactions to closely mimic laboratory conditions. No-penetration and no-slip boundary conditions were prescribed at the cylinder surfaces and the side walls. A velocity–inlet boundary condition was prescribed upstream of the cylinders, and a free boundary condition downstream. The inlet Reynolds number (*Re*) was set to 10,000 to produce the co-shedding regime, which has been shown to be stable in the *Re* which were ultimately selected for fish behavior experiments (*Re*=3700–12,400 (Alam et al., 2018; Sumner, 2010; Xu and Zhou, 2004).

The CFD model was validated by comparing the Strouhal number (*St*) and the instantaneous wake wavelengths of vortex streets with the experimental results obtained by Stewart et al. (2016). *St* was obtained from the dominant frequency of a Fast Fourier Transform (FFT) of the vertical component of the velocity vector (**U***_y_*) with respect to time. The instantaneous wake wavelength was determined by the spatial wavelength of **U***_y_* along the cylinder axis, extending downstream of the cylinders. The instantaneous wake wavelength was computed at each time step using the FFT of **U***_y_* with respect to the *x*-axis behind the cylinder. A grid convergence study was also conducted to ensure that numerical results would be independent of mesh size, time step and the computational domain.

The average vortex shedding frequency (*f*) for each of the two selected arrays at 74 cm s^−1^ was based on the *St* and physical parameters of the experimental tank by the following equation:
(1)

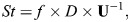
whereby *f* was solved for in terms of individual cylinder diameter (*D*) and the flow velocity in the region of the cylinders (*U*). To account for solid blocking effects causing flow constriction near the cylinders, *U* was calculated by:
(2)

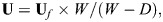
where *W* is the width of the tank and **U***_f_* is the nominal flow speed.

#### Optimization

Three vortex shedding regimes observed in tandem semi-circular cylinder configurations include the bluff-body regime (*L*/*D*<0.7), the reattachment regime (1.1<*L*/*D*<1.8) and the co-shedding regime (*L*/*D*>1.8) (Stewart et al., 2016). To ensure that there would be vortex formation between cylinders, we sought the optimal cylinder array spacing ratios that lie within the co-shedding vortex regime. An optimization procedure was thus performed to find the cylinder arrangement that maximizes Kármán vortex street periodicity within this regime because of the prevalence of Kármán gaiting in highly periodic wakes (Akanyeti and Liao, 2013a; Stewart et al., 2016). Sixty simulations were run to determine the optimal values of the design variables. Because of the large number of design variables and the non-linear nature of the dynamics, we sought an efficient optimization method. We employed surrogate based optimization (SBO) to approximate the objective function in the entire parameter space starting from 55 numerical simulations, called sample points (Adams et al., 2020). In this case, the objective function was the L-kurtosis of the FFT spectrum peakedness, which is periodicity.

In our optimization procedure, an initial set of random sample values of the design variables were first created to describe the design space. The design variables for this specific problem are the spacing parameters *L_x_*/*D* and *L_y_*/*D*. Following this, evaluations of the objective function on each sample point were performed in OpenFOAM. These evaluations were subsequently used to build the surrogate model upon which the optimal value could quickly be computed via an interpolation method. DAKOTA (Adams et al., 2020) was used to provide an interface between CFD (OpenFOAM) and analysis methods (SBO). Thirty simulations were selected out of the original set of 60 for exhibiting the co-shedding regime (Fig. 1A).

### Particle image velocimetry

The flow behind the KVS array and the PVS array, identified through numerical optimization, was experimentally validated using 2D PIV. 2D PIV is an imaging-based technique that measures the distribution of planar velocity components. The PIV measurements were conducted in a closed-loop water flume with dimensions of 30 cm height×25 cm width×89 cm in length. A 3D-printed honeycomb served as a flow straightener upstream of the working area in the flume.

The frame of the cylinder array was modeled in SolidWorks (version 2020) (Fig. 2A; Fig. S1D). The design was then manufactured out of clear acrylic to minimize visual stimuli for fish (Liao, 2006) by the UF Infinity Fab Lab (College of Design, Construction, and Production, University of Florida, Gainesville, FL, USA). D-shaped clear acrylic cylinders of 1.9 cm diameter were purchased separately. This frame allowed cylinders to be independently adjusted at varying lateral and streamwise distances, such that they could be disassembled and reconstructed to produce new arrangements (Fig. S1). The arrays were constructed within ±1 mm of the CFD simulations (Fig. 2B; Fig. S1A–C). Assembled arrays were lowered into the flow tank and secured to resist lift from the flow.

PIV measurements were taken on a plane extending in the streamwise and lateral directions, intersecting the cylinders near their mid-span to minimize end effects. A pair of front-surface mirrors were used to align the laser sheet, ensuring it was perpendicular to the flume wall. The field of view primarily focused on the flow downstream of the cylinders, where 20 continuously shed vortices were recorded behind the downstream cylinder for each experimental setup.

The flow field was illuminated by a 0.5 mm thick horizontal laser sheet, expanded from a 5 W continuous argon-ion laser beam (wavelength 532 nm, LaVision, Göttingen, Germany). A high-speed camera (frame rate 1000 frames s^−1^; resolution 1280×800 pixels; Miro LAB340, Vision Research, Wayne, NJ, USA) was used to record flows which were later edited in Phantom Camera Control 3.5 software and saved as AVI files. The camera was positioned in front of a mirror angled toward the clear bottom of the flume. The flow was seeded with silver-coated hollow glass sphere particles (diameter ∼14 μm; specific gravity 1.6; Potter Industries, Valley Forge, PA, USA). The field of view (150×90 mm) was recorded by a separate high-speed camera (frame rate 400 frames s^−1^; resolution 1280×800 pixels; Phantom Miro 310, Vision Research) for the selected speed 74 cm s^−1^ (*Re*=12,400). The frame rate was varied to ensure particle displacement of 5–8 pixels.

Because this was a 2D PIV experiment, a ruler was used for calibration. However, a calibration rig was constructed to ensure the ruler remained flat and aligned with the laser sheet during calibration. The particle images were enhanced and filtered (Roth and Katz, 2001) before being used to calculate velocity maps through a multipass cross-correlation technique, utilizing a commercial software package (LaVision^TM^ Davis). The cross-correlation process began with a coarse window of 48×48 pixels with 50% overlap, followed by a final pass using a 24×24 pixel window with 50% overlap, achieving a vector resolution of 1.433 mm.

To validate the Navier–Stokes equations used for simulation, we quantitatively compared the Strouhal number for vortex shedding by substituting the inlet flow velocity, **U**_∞_:
(3)

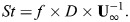


### Fish experiments

#### Trout acquisition and husbandry

A large number of juvenile rainbow trout (*O. mykiss*) were obtained from Wolf Creek National Fish Hatchery (Jamestown, KY, USA) to offer a large pool of subjects that could be drawn from in order to increase the likelihood of acquiring enough healthy fish of similar size. They were held in a 500 l recirculating round tank maintained at 15±1°C using an in-line chiller (Delta Star chiller, model DS-4-TXV, 106 Aqua Logic, San Diego, CA, USA). A continuous flow was created to accustom animals to swimming against current. Trout were allowed to acclimate to this environment for a minimum of 48 h before experimentation. Five individual rainbow trout were randomly selected for experimental trials (mean±s.e.m. body length 7.4±0.1 cm) and screened for fitness.

After data were collected for the first cylinder array, the fish was placed into a separate holding tank for the remainder of the study to ensure that it could be identified for subsequent experiments. Holding chambers were constructed out of 30 cm long PVC piping with netting, suspended in the main holding tank with other fish, and fish were fed a maintenance diet. This step was taken to avoid moving the cylinder arrays mounted in the flow tank, which could alter the hydrodynamics of the wake by disrupting their precise configuration. All experimental procedures were approved by the University of Florida Institutional Animal Care and Use Committee (IACUC) (ID 202200000056).

#### Fish swimming experimental conditions

Fish swimming experiments took place in the same 175 l recirculating flow tank as in the PIV experiments, maintained at 15±1°C (Stewart et al., 2016). A high-speed camera (100 frames s^−1^ at 1440×2560 pixel resolution, Miro LAB340, Vision Research) was aimed at a 45 deg mirror to image the ventral view of the flow tank. An LED light source (Lyra DMX, IkanCorp, Houston, TX, USA) placed above a white Plexiglas sheet was used as a diffuser to create a high-contrast silhouette of the fish.

Rainbow trout were exposed to the KVS array, the PVS array and a control treatment of freestream flow (no cylinder array) at each of three flow speeds (22, 48 and 74 cm s^−1^). The lowest velocity represents the flow speed at which trout were introduced into the flow tank. The highest flow speed was experimentally determined to be the fastest rate at which fish would hold station. The intermediate flow speed was selected for a third point of comparison. The *Re* of these flow speeds spanned the range 3700–12,400.

#### Experimental procedure

Individual trout were transported from the holding tank and introduced downstream to the cylinder array with the flow tank set at the lowest velocity treatment (Fig. 2C,D). A fixed, rectangular ‘zone of interest’ was determined from the development of vortices in vorticity contours generated by CFD simulations models. This zone occupied a certain downstream distance from the last column of cylinders and was used to guide the collection of video sequences for subsequent analysis of swimming kinematics (Fig. 2D). If a swimming fish did not hold station and drifted back against the downstream baffle, it was returned to its holding chamber to rest and experiments were continued at a later time. Sixteen total tail beats were collected from each individual for each flow speed and hydrodynamic treatment.

Upon completion of the experiment, trout were euthanized by an overdose of MS-222 and total length was measured. The anteroposterior COM was also determined by iteratively balancing the fish between two probes and measuring the balance point (Liao et al., 2003a).

#### Digitization of fish behavior

**Fig. 3.**
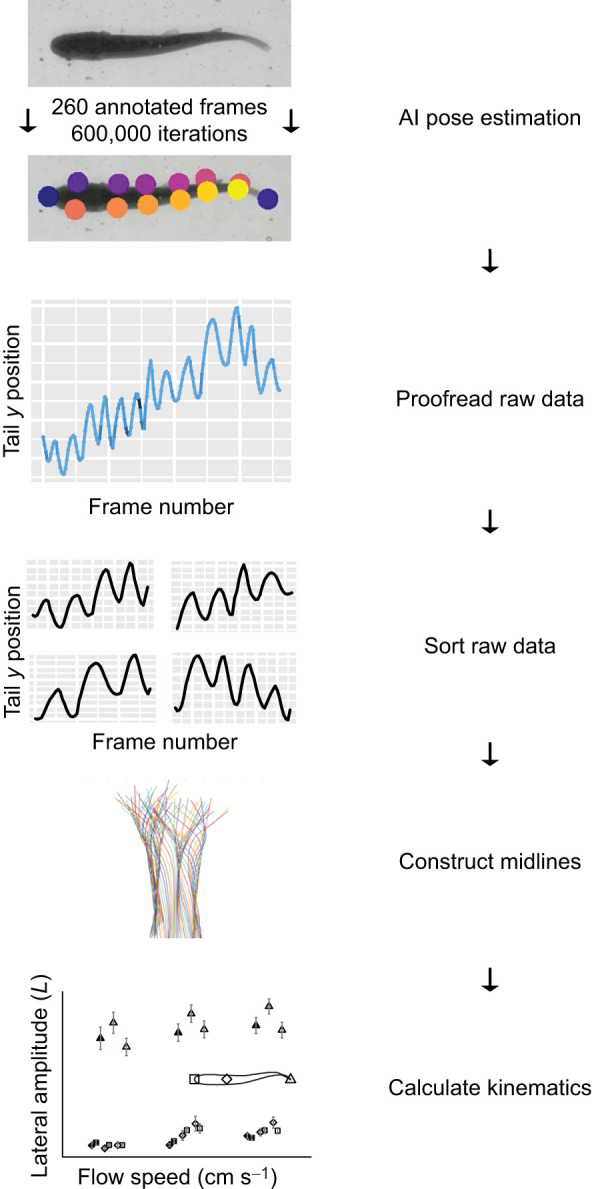
**Schematic concept of the computational workflow.** Annotations of the snout, tail tip and 6 points along both sides of the fish's body were placed on 260 representative frames in DeepLabCut (ipython). A library was made by training DeepLabCut on those frames for 600,000 iterations. DeepLabCut then estimated the annotated body positions on all video data. These estimations were manually proofread for extreme, obvious miscalculations by DeepLabCut, then sorted into sets of four tail beats (R). The midlines of the fish were constructed, and thence kinematics were calculated (MatLab).

The body silhouette of each fish was identified using DeepLabCut (version 2.1.10.1) (Mathis et al., 2018; Nath et al., 2019) with ipython (version 7.20.0) (Fig. 3). Training data were developed from 260 frames in which the snout, tail tip and six points on the left and right side of the fish outline were annotated in varying luminance and noise (i.e. bubbles). The data were trained for 600,000 iterations before predicting locations on the remaining data. Predicted locations were graphically and visually inspected for gross anomalies using a custom script in R (version 4.0.4). Data were then manually grouped into sequences of four tail beats in R. A midline was then reconstructed with a modified version of the custom script ‘Clickdigfish_matlab2015a’, and body kinematics were calculated with the custom script ‘PlotFish’ (MatLab version R2020a; Liao et al., 2003a). If >10% of data from a video file were inaccurate, the outline of the fish was manually digitized with the unmodified script.

Tail beat frequency, body wavelength and the amplitude at three locations along the body (snout, COM and tail tip) were calculated using MatLab. Tail beat frequency was determined from the inverse of the average period (time between maxima and minima of tail beats). Wavelength was calculated as the average phase speed (determined from the mean speed of maxima traveling along the midline) divided by the tail beat frequency. All amplitudes were calculated by halving the distance between maxima and minima along the midline (i.e. maximum lateral excursion from the midline).

#### Statistical analysis

Amplitude data were log transformed to satisfy normality. One datum was an outlier and removed from the snout amplitude data because it was clear from reviewing the video that the fish was not holding station, and one set of four tail beats was removed for the same reason. All statistical analyses were conducted in R (https://CRAN.R-project.org/package=rstatix). We calculated means of kinematics with s.e.m. (Table 1), then ran two-way ANOVA with a false discovery rate (FDR) adjustment to compare hydrodynamic treatments and flow speeds. Where significance was found, we conducted Tukey's HSD *post hoc* tests (Tukey–Kramer HSD in the case of the snout amplitude) at 95% confidence to determine differences by either main effect or interaction effect. We further conducted one sample, left-tailed *t*-tests to compare the mean tail beat frequencies of both cylinder arrangements at 74 cm s^−1^ with their respective vortex shedding frequencies.

**Table 1.**
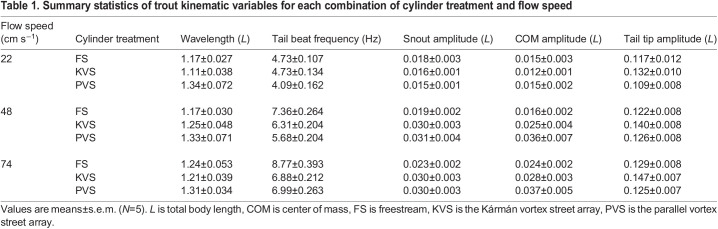
Summary statistics of trout kinematic variables for each combination of cylinder treatment and flow speed

## RESULTS

### CFD and optimization

The goal of the CFD simulations was to identify cylinder arrangements that optimally generate periodic Kármán vortex wakes within and downstream of a 3×5 cylinder array. The objective function, L-kurtosis, was maximized within this optimization, which quantifies the periodicity of Kármán vortex streets. This analysis gives an improved understanding of the influence of the spacing ratio in the streamwise (*L_x_*/*D*) and cross-stream (*L_y_*/*D*) directions on Kármán vortex street formation.

The interplay between the streamwise and cross-stream spacing ratios was responsible for achieving optimal Kármán vortex streets within the co-shedding regime, such that there was no single optimal spacing ratio. Instead, a distribution within the parameter space conveyed a range of values for *L_x_*/*D* (1.9–2.3) and *L_y_*/*D* (2.4–3.5) that can promote the formation of optimized Kármán vortex streets (Fig. 1A).

In this optimal range, vortical structures generated between cylinders hold their shape as they translate downstream. An alternating vortex shedding pattern forms, and the subsequent Kármán vortex street formation downstream of the arrays also preserves its shape with high periodicity. We selected a Kármán vortex street from this range with a Strouhal number of 0.21 and a periodicity of 0.1059 m^2^ s^−1^ (L-kurtosis=0.0063) (*L_x_*/*D*=1.9, *L_y_*/*D*=3.0; KVS array; Fig. 1B). Outside this range, the large vortical structures generated from the most upstream cylinders failed to maintain their shape as they translated downstream. This phenomenon is likely due to the improper impingement of the vortical structures on the downstream cylinders, thus causing a disrupted flow field downstream of the array. Hence, vortex street formation downstream of the arrangements that were outside the optimal range were lacking in periodicity. We selected a flow field with similar features to a parallel vortex street (Karasudani and Funakoshi, 1994) in which a symmetrical vortex street forms and vortices were separated cross-stream from the center cylinder's streamwise axis. The Strouhal number was 0.23 and the periodicity was 0.00557 m^2^ s^−1^ (L-kurtosis=5.39×10^−5^) (*L_x_*/*D*=2.6, *L_y_*/*D*=2.2; PVS array; Fig. 1C). Periodicities for both arrays were greater than 3×10^−5^ m^2^ s^−1^, the maximum value considered by a previous study looking at fish swimming behind two cylinders (Stewart et al., 2016).

### Validating the numerical simulations

**Fig. 4.**
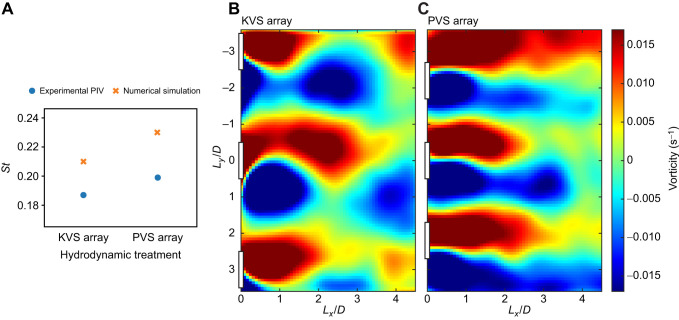
**Strouhal number comparison between particle image velocimetry (PIV) and CFD results alongside vorticity contours for experimental validation of CFD.** (A) Strouhal numbers (*St*) show good agreement between experimental and simulation results. Instantaneous vorticity contours show (B) an alternating vortex shedding pattern, similar to the KVS array observed via CFD and (C) a symmetric vortex shedding pattern, similar to the PVS array observed via CFD. Vorticity contours show vorticity as a color gradient graphed onto *L_x_*/*D* versus *L_y_*/*D* space. Locations of the D-shaped cylinders are represented by rectangles on the *L_y_*/*D* axis.

Both the quantitative and qualitative results from the PIV indicate good agreement with the results of our selected CFD simulations. The experimental Strouhal number was 0.18 in the KVS array and 0.2 in the PVS array (Fig. 4A). This shows that our simulations accurately predict the frequency of vortex shedding. Qualitatively, the cross-stream velocity contours and instantaneous vorticity contours from the PIV experiments matched well with those from the numerical simulations: the KVS array exhibited alternating vortex shedding downstream (Fig. 4B), while the PVS array exhibited symmetric vortex shedding (Fig. 4C).

This validation confirms that our simulation results are consistent with the flow fields selected for fish swimming experiments. It indicates that our simulations can reliably replicate experimental conditions and results, providing confidence in their accuracy for interpretation of fish behavior.

### Swimming kinematics across hydrodynamic treatments and flow speeds

**Fig. 5.**
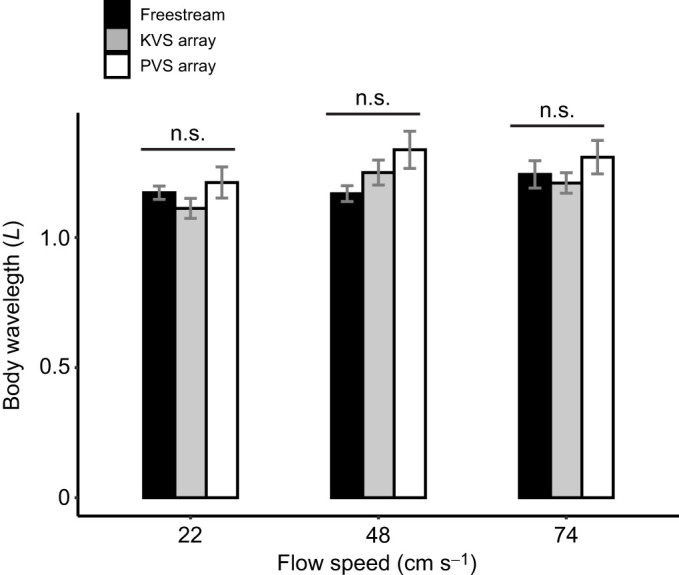
**Mean body wavelength across flow speeds and hydrodynamic treatments.** Data are means±s.e.m. (95% error), *n*=5 fish per treatment combination, where shade indicates hydrodynamic treatment (freestream, Kármán vortex street and parallel vortex street) and *L* is fish body length. A false discovery rate (FDR)-adjusted two-way ANOVA detected a main effect on wavelength due to hydrodynamic treatment (reported in Results), but no significance (n.s.) was found by a Tukey's *post hoc* test.

Neither flow velocity (*F*=3.02, *P*=0.0514) nor the interaction term with hydrodynamic treatment (*F*=0.676, *P*=0.6098) had a significant effect on body wavelength after FDR adjustment (α=0.0333; Fig. 5). Hydrodynamic treatment did have a main effect on wavelength (*F*=3.54, *P*=0.0312), although no significance was found in the *post hoc* test. Conversely, there was a significant interaction effect between hydrodynamic treatment and flow velocity on tail beat frequency (*F*=4.527, *P*=0.00168). Both flow velocity and hydrodynamic treatment had main effects on snout amplitude (*F*=24.972, *P*=3.1×10^−10^; *F*=4.166, *P*=0.0171, respectively) and COM amplitude (*F*=23.003, *P*=1.42×10^−9^; *F*=4.495, *P*=0.0125, respectively), though the effect of hydrodynamic treatment was smaller. Flow velocity (*F*=3.987, *P*=0.0203) and hydrodynamic treatment (*F*=5.79, *P*=0.00368) also had an effect on tail tip amplitude, but hydrodynamic treatment had a greater effect than flow velocity, which was the opposite pattern to the anterior body amplitudes. The interaction effects on snout (*F*=1.995, *P*=0.0975), COM (*F*=1.682, *P*=0.1564) and tail tip (*F*=0.124, *P*=0.97358) amplitudes were not significant.

**Fig. 6.**
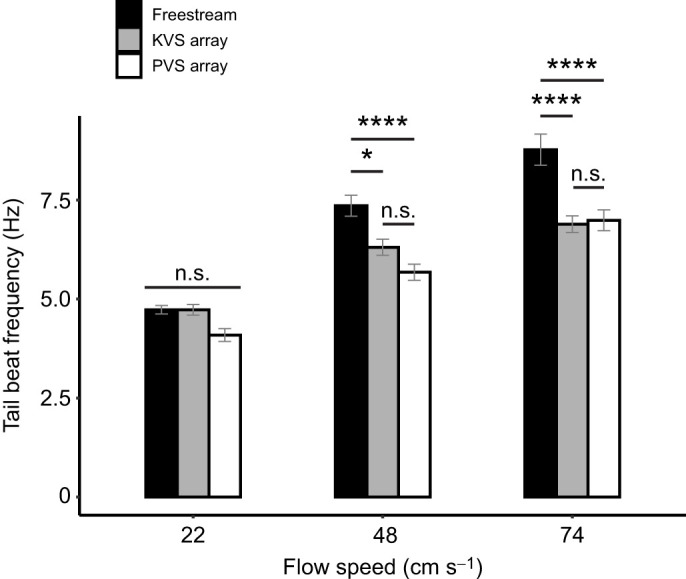
**Mean tail beat frequency across flow speeds and hydrodynamic treatments.** Data are means±s.e.m. (95% error), *n*=5 fish per treatment combination, where shade indicates hydrodynamic treatment (freestream, Kármán vortex street and parallel vortex street). Tukey's adjusted significance levels according to Tukey's *post hoc* test following a FDR-adjusted two-way ANOVA are represented by asterisks (****P<0.0001, *P<0.05; n.s., not significant). Also notable, frequency differed significantly (*P*<0.0001) between each flow speed and either of the other flow speeds within each hydrodynamic treatment, except for between 48 and 74 cm s^−1^ in the Kármán vortex street.

Tail beat frequency generally increased with flow velocity, though it was generally lower behind cylinder arrangements than in the freestream (Tukey's test: α=0.05; Fig. 6). Within hydrodynamic treatments, there were differences between each velocity and either other velocity, except within the KVS array between 48 and 74 cm s^−1^. There were no significant differences between hydrodynamic treatments at 22 cm s^−1^. At 48 cm s^−1^, the tail beat frequency in the freestream was significantly higher than that in the PVS array (*P*=2.48×10^−5^) and the KVS array (*P*=0.0398) but did not differ significantly between the PVS and KVS arrays. Similarly, at 74 cm s^−1^, the tail beat frequency was significantly higher in the freestream than in both the PVS (*P*=7.08×10^−6^) and the KVS array (*P*=1.66×10^−6^), but did not significantly differ between the PVS and KVS arrays. This points to an increasing tail beat frequency with velocity, but an overall lower rate of increase when trout swam downstream to the multiple cylinder array compared with the freestream.

Tail tip amplitude differed most significantly between the PVS array and the KVS array (*P*=0.00618). There was also a significantly larger tail tip amplitude in the KVS array than in the freestream (*P*=0.018), but the amplitude did not significantly differ between the PVS array and the freestream. Among velocities, there was one significant difference in tail tip amplitude between the lowest and the highest flow speeds (*P*=0.0202), and no other significant differences. Overall, it seems that the KVS array had the greatest effect on the tail tip amplitude.

**Fig. 7.**
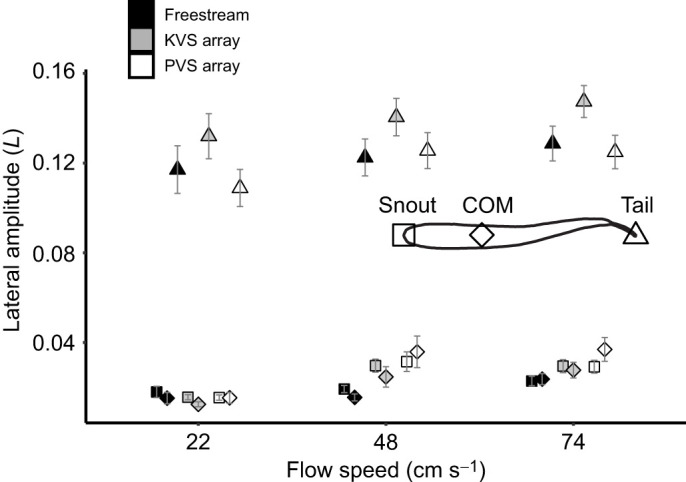
**Mean lateral amplitude across flow speeds and hydrodynamic treatments for three different locations along the body.** Data are means±s.e.m. (95% error), *n*=5 fish per treatment combination, where shade indicates hydrodynamic treatment (freestream, Kármán vortex street and parallel vortex street), shape represents body location [square, snout; diamond, center of mass (COM); triangle, tail tip] and *L* is fish body length. Results of a FDR-adjusted two-way ANOVA with a Tukey's *post hoc* test are reported in Results.

The most significant increases in COM and snout amplitudes were between the lowest flow speed and the two highest speeds (e.g. maximum *P*=9.20×10^−5^ between 22 and 48 cm s^−1^ for the COM) (Fig. 7). The COM amplitude also differed significantly between the two highest speeds (*P*=0.0438), but the snout amplitude did not. Among hydrodynamic treatments, the COM amplitude only differed significantly between the freestream and the PVS array (*P*=0.00886). The snout amplitude, however, differed significantly between the freestream and both the PVS (*P*=0.0434) and the KVS array (*P*=0.0296). Though the interaction terms of the ANOVA were non-significant, it is qualitatively apparent from Fig. 7 that variation between hydrodynamic treatments was more pronounced at the two highest speeds than at the slowest speed. Minimally, both velocity and the cylinder arrangements affected amplitudes on the anterior body.

The vortex shedding frequencies of the KVS and PVS arrays at 74 cm s^−1^ were 8.85 Hz and 9.69 Hz, respectively. The tail beat frequencies in both cylinder arrangements were significantly lower than their respective vortex shedding frequencies (KVS array: *P*=8.931×10^−9^; PVS array: *P*=1.679×10^−9^).

### Qualitative observations of swimming behaviors

**Fig. 8.**
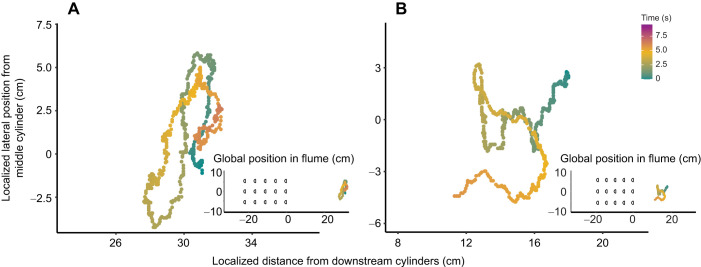
**Cases of casting behavior in rainbow trout illustrated by digitized snout position across time.** All *x*–*y* axis units are 1:1. These instances are at a flow speed of 48 cm s^−1^ for (A) the parallel vortex street and (B) the Kármán vortex street. Inset graphs show corresponding global position to the multiple cylinder array.

**Fig. 9.**
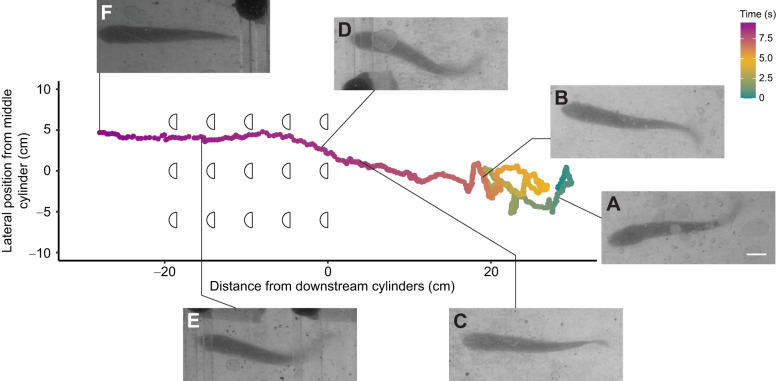
**A representative, continuous recording of a casting event followed by acceleration through the cylinder array for a single fish in a Kármán vortex street at 74 cm** **s^−1^.** *x*–*y* axis units are 1:1 and time scale is the same as in Fig. 7. Images (A–F) are zoomed-in video frames of the trout swimming at specific time points indicated by the lines that connect the graph to the panels. Scale bar in A: 1 cm (applies to all panels). (A,B) Casting behavior, in which the trout angles its body against the flow and swims left or right in the flume. (C) Following casting, the trout swims quickly and directly to the middle downstream cylinder. The trout lingers and appears to entrain behind the cylinder edge for a moment. (D) The trout accelerates into the cylinder array and (E) continues to swim through the array with the same swimming movement, in this case briefly pausing twice. (F) Once past the cylinder array, the trout shows a similar behavior to that in C, though the position would imply bow waking. The trout resumes a plethora of behaviors shortly after F (observed out of camera view).

Though we set out to analyze station-holding behaviors, we observed other behaviors such as forward acceleration, entraining, wall following and vertical movements through the water column. Among these behaviors were lateral trajectories we refer to as ‘casting’. When we increased flow velocity, trout made successive sweeping lateral excursions, sometimes spanning the entire flume (Fig. 8). This was followed by swimming directly to the downstream edge of the cylinder array, where trout would momentarily entrain behind a single cylinder before accelerating through the entire cylinder array (Fig. 9). Once upstream of the cylinder array, it swam with similar motions to bow waking, yet unlike bow waking it held station upstream of the gap between cylinders rather than directly in front of a cylinder.

## DISCUSSION

### Hydrodynamics behind cylinder arrays

CFD simulations revealed known flow patterns which have not been shown to be produced by D-shaped cylinders arranged in a 3×5 pattern. Unlike for a single cylinder where the wake contains vortices that have a similar diameter to the cylinder, our results support recent analyses which show that it is possible for vortices in the wake of multiple cylinders to be larger in diameter than any single cylinder (Gao et al., 2020). In the KVS array of this study, the drag wake behind the central cylinder draws in vortices from lateral cylinders, which circulate in the same direction as the vortices behind the central cylinder. Holding the streamwise cylinder spacing constant and increasing the cross-stream spacing results in the opposite effect, instead producing dissipated vorticity behind the central cylinder and coherent lateral vortex streets.

After experiments were conducted, we calculated the turbulent kinetic energy (TKE) of all CFD simulations directly with OpenFOAM's built-in program (Fig. S2). The TKE of the KVS and PVS array was 0.10453 m^2^ s^−2^ and 0.07076 m^2^ s^−2^, respectively. The range of cylinder spacings which were optimal for periodic Kármán vortex streets (including the KVS array) also possessed higher TKE than most other cylinder spacings, likely as a result of the presence of coherent vortices. In the PVS array, the flow field was characterized by dissipated vortices and the TKE was correspondingly lower.

### Swimming kinematics of trout behind cylinder arrays

#### Body undulation while holding station in a vortex street and freestream

Swimming trout hold station in steady flows by passing a mechanical body wave from head to tail with increasing lateral amplitude (Bainbridge, 1962; Di Santo et al., 2021; Webb, 1988). In the presence of a coherent Kármán vortex street, these kinematics change significantly, and fish adopt a Kármán gait (Liao et al., 2003a,b). This mode of locomotion occurs when the length of the fish is 2–4 times the diameter of the shed cylinder vortices. The Kármán gait is identifiable by large lateral amplitudes across the body, a longer body wavelength, and a decreased tail beat frequency that is slightly higher than the vortex shedding frequency (Liao et al., 2003a; Akanyeti and Liao, 2013a; Stewart et al., 2016). Kármán gaiting fish slalom between vortices as they exploit each vortex's energy in a largely passive way, with little to no axial muscle activity (Liao, 2007; Liao et al., 2003b; Taguchi, 2011). A model of Kármán gaiting reveals that it is freestream swimming kinematics superimposed onto a vortex street, which generates greater lateral body translations, body rotations and head yaw motions (Akanyeti and Liao, 2013b).

We expected that fish would Kármán gait in the KVS array, but their station-holding kinematics did not meet the criteria for Kármán gaiting (Akanyeti and Liao, 2013b). Kármán gaiting fish synchronize their tail beat frequency with the cylinder vortex shedding frequency, but in this study the tail beat frequency was lower than the expected vortex shedding frequency behind both cylinder arrangements (Akanyeti and Liao, 2013a; Liao et al., 2003a). The body wavelength did not significantly differ between any treatments, including the KVS array and freestream (Fig. 5). The snout, COM and tail tip amplitudes were also lower than that of Kármán gaiting fish (less than 80%, 80% and 50%, respectively; Akanyeti and Liao, 2013a; Liao et al., 2003a). We suggest that the absence of large lateral translations of the body indicates that fish are swimming through, rather than slaloming between, cylinder vortices. Overall, the kinematics of trout holding station behind our cylinder arrays are similar to freestream swimming, to the point where swimming movements could be considered modifications of freestream swimming rather than movements resembling Kármán gaiting.

One possible reason for the absence of Kármán gaiting is that vortices were not as coherent as expected in the zone of interest where we collected kinematics data. Maintaining trout in one location for long enough periods of time to record their swimming motions was often challenging because trout commonly casted (Fig. 8). Additionally, if a trout was close enough to the cylinder array, it typically accelerated through the array (Fig. 9), which is why the zone of interest was distanced from the downstream cylinders. As vortex strength diminishes with distance from a bluff body (Tritico and Cotel, 2010), the strength of the wake may have diminished in the zone of interest to a level where the trout would not Kármán gait.

Another explanation involves the organization and morphology of vortices. A fish in a Kármán vortex street ‘slaloms’ between vortices, where there is adequate space between vortices in both the streamwise and cross-stream directions for fish to swim. The streamwise spacing of vortices in the KVS array of this study was comparable to that of other studies in which fish Kármán gait (Liao et al., 2003a,b; Stewart et al., 2016). In the cross-stream direction, however, there was no space between vortices because of their oblong morphology and because vortices align on a streamwise axis behind the central cylinder rather than stagger (Fig. 1B).

It is also possible that the lack of differences between hydrodynamic treatments in general is due to low statistical power. We selected a conservative sample size of individuals because of the intensive multidisciplinary nature of combining computational exploration with live fish swimming kinematics. Studies on fish swimming kinematics often use a similar number of individuals to the present study, especially when they involve examining parameters of the fluid (Jayne and Lauder, 1995; Liao et al., 2003a; Lucas et al., 2020; Stewart et al., 2016; Tytell, 2004), focusing on controlling for variation between tail beats or video segments. Although we compared 80 tail beats per treatment and controlled for fish length, it is possible that the low number of individuals presented a confounding factor which impeded our ability to discern certain statistical differences.

#### Comparison of swimming kinematics across cylinder arrays

Despite potentially large differences in wake periodicity and TKE, only tail amplitude increased significantly in the KVS array compared with the PVS array and freestream treatments. This pattern is similar to Kármán gaiting fish in a vortex street, though the amplitudes in the Kármán gait are relatively larger (Akanyeti and Liao, 2013a,b; Liao et al., 2003a; Stewart et al., 2016). In the KVS array, vortices are in line and TKE values are high, thereby creating a condition in which fish can intercept stronger, successive vortices of alternating circulation. The PVS array, in contrast, has low TKE and vortices that translate symmetrically downstream in parallel, distanced from the middle of the flume; in effect, PVS array vortices might be said to weakly graze a fish's body rather than intercept it.

It is unclear whether the higher tail amplitude in the KVS array is due to passive buffeting or actively controlled by muscular activity. Tail muscles have been shown to be inactive in Kármán gaiting (Liao, 2004), and even a dead trout will exhibit an increased tail amplitude (Beal et al., 2006), so it is possible that the tail is buffeted passively in the KVS array. However, the fishes' overall swimming kinematics in this study best resemble a fish swimming in a freestream, which requires sequential muscle activity (Altringham and Ellerby, 1999; Gillis, 1998; Hammond et al., 1998; Jayne and Lauder, 1995; Liao, 2004; Wardle et al., 1995). Furthermore, a dead trout (Beal et al., 2006) towed in a Kármán vortex street passively synchronizes its tail beat frequency to the vortex shedding frequency, whereas the tail beat frequency of the present study was significantly less than the vortex shedding frequency. This indicates a level of muscular control, whether to increase the tail amplitude or to prevent the tail amplitude from further increasing. Both mechanisms could also offer interesting implications for vortex exploitation given that work on both passively oscillating foils (Wu and Chwang, 1975) and harmonically oscillating foils (Triantafyllou et al., 2000) has shown the capacity for vorticity control and thrust production in a vortex street. Regardless, the proximity of strong, successive vortices in the KVS array appears to play an important role in tail beat amplitude and indicate some level of muscular control that is not present in the PVS array.

The COM amplitude in a swimming fish increases with speed (Xiong and Lauder, 2014), and can play an important role in stability (Lauder and Drucker, 2004; Webb and Weihs, 1994). We found that the COM amplitude was also greater in the PVS array than in the freestream, yet did not significantly differ in either hydrodynamic treatment from the KVS array. These amplitudes may reflect the lateral translation of the body through the interaction with vortices rather than increases in body wave amplitude during undulation as there were no differences in wavelength among hydrodynamic treatments. Translational movements of the COM likely have little impact on swimming stability (Webb, 2002), while large lateral translations can help to exploit vortex recapture during Kármán gaiting (Liao, 2004; Liao et al., 2003a).

The lower periodicity of vortical flows behind tandem cylinders is less predictable to fish and can be punctuated by more chaotic flow (Stewart et al., 2016; Igarashi, 1981). Given the low TKE of the PVS array, it is likely that these chaotic flows accumulate below the integral scale, as the majority of vortices in turbulence are small (Tritico and Cotel, 2010). This combination of low periodicity and low TKE in the PVS array therefore produces a subtle and less predictable flow, which may affect control of the COM. If correct, it could explain the kinematic difference seen in the PVS array from the freestream, even though the COM amplitude in the KVS array does not differ from either the PVS array or the freestream.

Compared with swimming in the freestream, tail beat frequency decreased and snout amplitude increased when cylinders were present, yet did not differ between cylinder arrays. Tail beat frequency was also lower than the expected vortex shedding frequency in both cylinder arrays. This suggests that trout did not synchronize their tail beats to vortices as has been observed when Kármán gaiting (Akanyeti and Liao, 2013b; Liao et al., 2003a). To investigate whether tail beat frequency was simply the result of swimming in the reduced velocity behind the cylinders, we calculated the tail beat frequency of a fish swimming in the freestream at the value of the reduced flow produced by each cylinder array. We did not observe a difference in the tail beat frequency between the reduced flow speed and the experimental array, indicating that the decreased tail beat frequency may be due to reduced flow. This would not be the case for the increased snout amplitude, however, because snout amplitude decreases with flow speed (Table 1). It is more likely, then, that variables of the wake itself affected the snout amplitude. While the mechanism is unclear in our experiment, lateral motions of the snout can produce thrust when brook trout (*Salvelinus fontinalis*) swim in a freestream (Lucas et al., 2020). It would be interesting to investigate the interactions of the anterior body with different unsteady flows to explore alternative methods of thrust production in cylinder wakes.

We expected that swimming kinematics behind cylinder arrays would differ from those for freestream swimming, but we did not expect that they would differ in similar ways between arrays because of the substantial differences in periodicity, TKE and vorticity. The underlying mechanisms remain unclear, but it is notable that (1) tail beat amplitude is the only kinematic which differed between cylinder arrays, (2) COM amplitude only differed between the PVS array and freestream, and (3) snout amplitude and tail beat frequency changed in opposite directions (increase versus decrease) in response to the presence of cylinders. Simultaneous visualization of fish movement and fluid dynamics or electromyography would provide valuable insight in future studies (Liao, 2004; Liao et al., 2003a,b; Stewart et al., 2016; Tritico and Cotel, 2010).

### Casting behavior

We commonly observed sweeping behaviors whereby previously station-holding trout swam in the cross-stream direction when exposed to higher flow velocities behind cylinder arrays (Fig. 8). These sweeping movements spanned tens of centimeters and were often followed by upstream acceleration through the cylinder array (Fig. 9). We hypothesize that sweeping allows trout to explore the source and bounds of their fluvial environment, similar to how moths cast to sample pheromone plumes (Kennedy, 1983). Wilkes et al. (2017) found that Atlantic salmon move quickly around their experimental arena to sample the flow. Cross-stream sweeping has also been described in Mexican tetras (Elder and Coombs, 2015) and giant danio (Bak-Coleman et al., 2013) in a variety of flow and sensory (lateral line, visual) conditions. Although this study focused on station-holding kinematics, the motivation of fishes in turbulence may be to escape upstream. Casting is a robust behavior that should be considered in future studies looking at the ability of fishes to navigate unsteady flows behind complex physical structures.

### Summary

We selected two cylinder arrays that exhibited turbulent flows of differing periodicity and vortex street organization using CFD modeling. We then fabricated these cylinder arrays and experimentally placed live fish behind them. Fish did not Kármán gait, but rather held station using swimming kinematics that more closely resembled undulation during freestream swimming. Even so, we did observe a greater tail amplitude in the KVS array, with implications for vorticity control. We also observed certain kinematics differing from freestream treatment owing to the effect of cylinders on the motion of the fluid, though not between cylinder arrays: namely, the COM amplitude increased in the PVS array and the snout amplitude increased in both the KVS array and the PVS array by similar amounts. It is possible that vortices were too dissipated in our zone of interest for fish to properly Kármán gait, or alternatively that the organization and morphology of the KVS array did not make Kármán gaiting feasible. Trout also commonly performed large, cross-stream searching motions before accelerating through the cylinder array, suggesting that sensory inputs can influence higher-order behaviors.

## Supplementary Material

10.1242/jexbio.247873_sup1 Supplementary information
