# Supplementary material for: Swimming kinematics of rainbow trout behind a 3×5 cylinder array: a computationally driven experimental approach to understanding fish locomotion
Source: J Exp Biol. 2024 Dec 5;227(23):jeb247873. doi: 10.1242/jeb.247873 (PMC11658682; doi:10.1242/jeb.247873)
Supplement: Supplementary information [file jexbio-227-247873-s1.pdf]

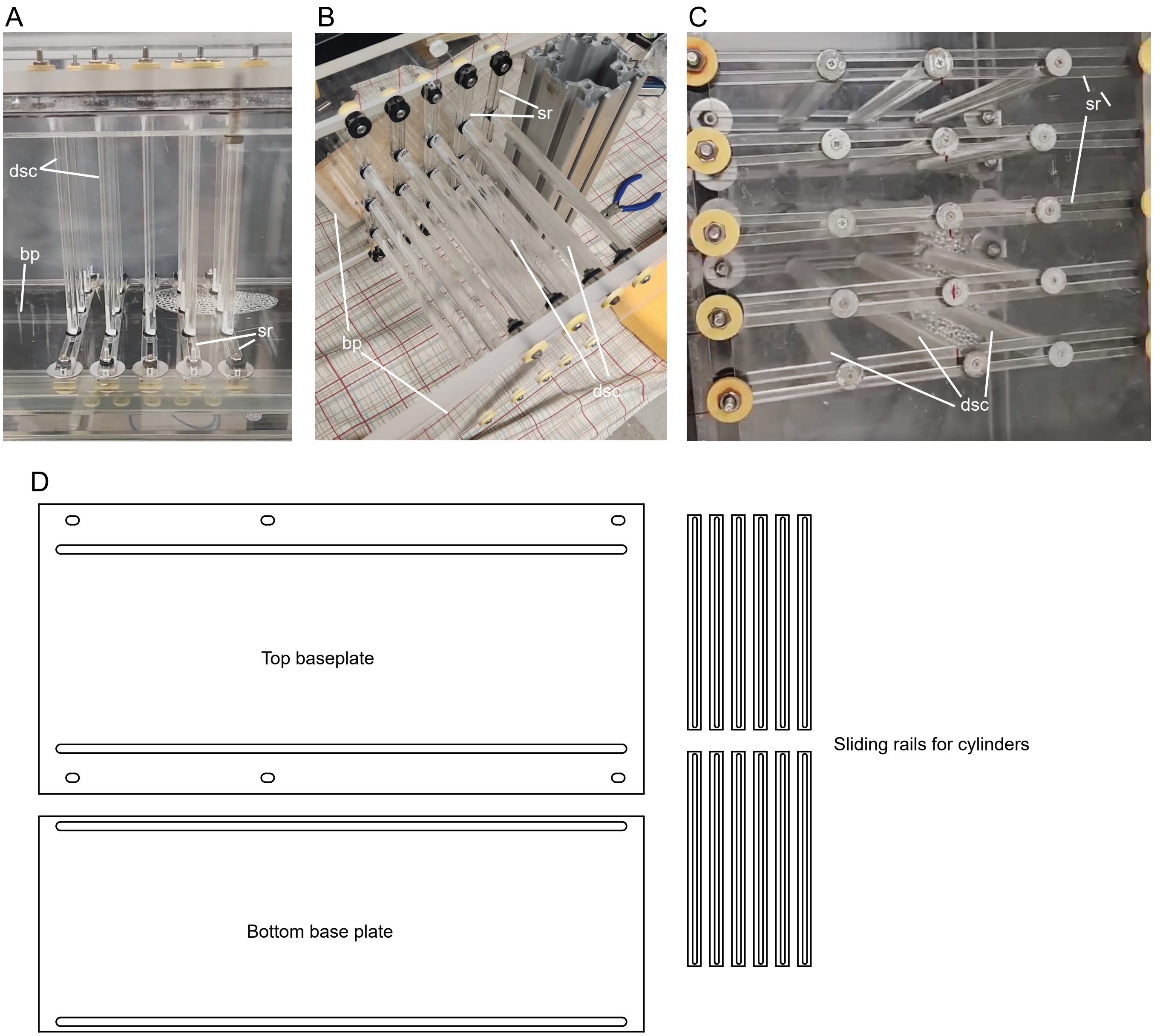

Fig. S1. Schematic used for laser cutting sheets of clear acrylic and representative images of fully constructed 3 x 5 cylinder array. D-shaped clear acrylic cylinders (i.e. half cylinders; diameter = 1.9 cm) were purchased separately. (A-C) Cylinders were secured to sliding rails with metal screws and circular washers. Sliding rails were secured to the top and bottom baseplates with metal nuts and bolts, and rubber washers. To baseplate was screwed into recirculating flow tank. Different images show (A) sideview (B) angled view (C) top view. (The contrast in A and C have been modified to highlight the cylinders due to dim lighting). (D) Sheet cut image with exact dimensions of cylinder array. See Figure 2 in the body of the manuscript for further information. (Key: dsc = D-shaped clinder, sr = sliding rail, bp = baseplate)

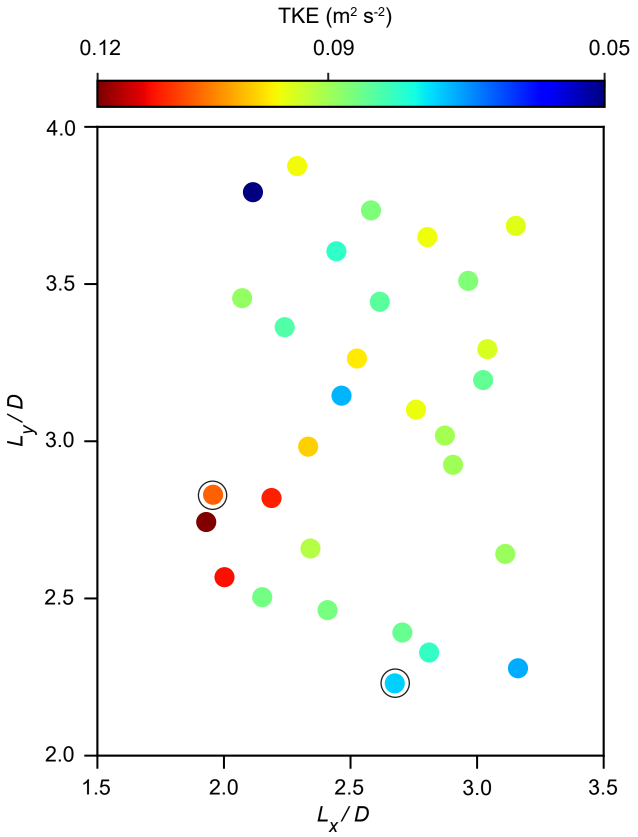

Fig. S2. Turbulent kinetic energy values corresponding to the spacing ratios selected from the periodicity optimization results that lie within the co-shedding regime. Color gradient scale represents TKE ($m^2 s^{-2}$) for each plotted cylinder spacing ratio (Lx/D and Ly/D). The KVS array possessed a relatively higher TKE than the PVS array (both are circled).

Journal of Experimental Biology • Supplementary information
